# Chronic granulomatous disease with pulmonary mass-like opacities secondary to hypersensitivity pneumonitis: a case report

**DOI:** 10.1186/1752-1947-8-242

**Published:** 2014-07-02

**Authors:** Yuki Katsuya, Masayuki Hojo, Shigeo Kawai, Toshinao Kawai, Masafumi Onodera, Haruhito Sugiyama

**Affiliations:** 1 National Center for Global Health and Medicine, Division of Respiratory Medicine, 1-21-1 Toyama, Shinjuku-ku, Tokyo 162-8655, Japan; 2 National Center for Global Health and Medicine, Division of Pathology, 1-21-1 Toyama, Shinjuku-ku, Tokyo 162-8655, Japan; 3 Department of Human Genetics, National Center for Child Health and Development, 2-10-1 Okura, Setagaya-ku, Tokyo 157-8535, Japan

## Abstract

**Introduction:**

Chronic granulomatous disease, one of the primary immunodeficiency syndromes, is characterized by failure of phagocytic capacity due to loss of reactive oxygen species production, as well as formation of granulomas in organs. Clinically, dysregulated inflammation by excessive cytokine production due to loss of reactive oxygen species production is suggested as a cause of noninfectious inflammatory problems such as chronic granulomatous disease colitis. We experienced a rare case of a patient with chronic granulomatous disease with unique pathological and radiological presentations of hypersensitive pneumonitis, which to our knowledge has never been previously reported.

**Case presentation:**

A 20-year-old Japanese man with chronic granulomatous disease was referred due to cough and abnormal chest imaging findings. Computed tomography of his chest showed diffuse, bilateral, centrilobular nodules and multiple mass lesions in lower lobes that do not fit a common image of hypersensitivity pneumonitis. Pathological findings of both nodules and mass lesions on surgical lung biopsy were homogeneous, and excessive granulomas in the bronchioles and alveolar duct as well as lymphocytic alveolitis were seen, all consistent with hypersensitivity pneumonitis. The radiological and laboratory abnormalities did not improve after antigen avoidance; however, they disappeared after high-dose steroid therapy.

**Conclusions:**

When we encounter a case of hypersensitive pneumonitis showing atypical pulmonary mass-like opacities in a patient with chronic granulomatous disease, we should consider hyperinflammatory status and excessive granuloma formation of chronic granulomatous disease and start with high-dose steroid therapy as treatment.

## Introduction

Chronic granulomatous disease (CGD) is a hereditary immunodeficiency disease characterized by failure of phagocytic capacity due to a defect of nicotinamide adenine dinucleotide phosphate reactive oxygen species production and lack of bacterial killing, which causes recurrent life-threatening infections. The mechanism for granuloma formation remains unclear, but the neutrophilic response persists for an abnormally long period at sources of inflammation and results in chronic inflammation. However, reactive oxygen species also regulate cell signaling by activating nuclear factor-κB and other transcription factors and the production of cytokines such as interleukin-1β and tumor necrosis factor-α, even in nonphagocytic cells. Clinically, dysregulated inflammation by excessive cytokine production due to loss of reactive oxygen species production is suggested as a cause of noninfectious inflammatory problems such as CGD colitis [1,2]. Here, we report unusual pathological and radiological presentations of hypersensitive pneumonitis in a patient with CGD, which have never been reported.

## Case presentation

A 20-year-old Japanese man was referred due to nonproductive cough and abnormal findings on chest imaging. His mother was a heterozygous carrier of CGD, and he was diagnosed as having X-linked recessive CGD lacking gp^91phox^ at the age of 1 year. He was given prophylaxis with trimethoprim-sulfamethoxazole and itraconazole and had been well for 19 years. When he was 19-years old, he developed CGD-related colitis and started treatment with prednisone 45mg per day. He started working at a warehouse 4 months later. After 3 months, a routine chest X-ray showed abnormalities. He then developed a nonproductive cough unresponsive to either antibiotics or antifungals.

**Figure 1:**
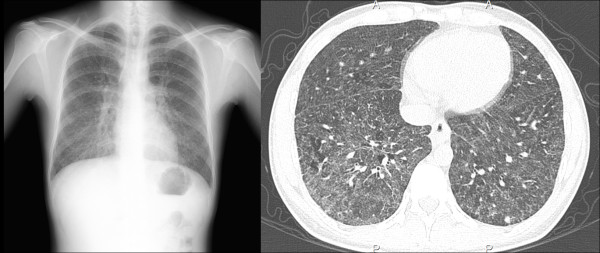
**Computed tomography of the chest.** Computed tomography of the chest shows diffuse bilateral fine centrilobular nodules, as well as some mass lesions in lower lobes that do not fit a common image of hypersensitivity pneumonitis.

**Figure 2:**
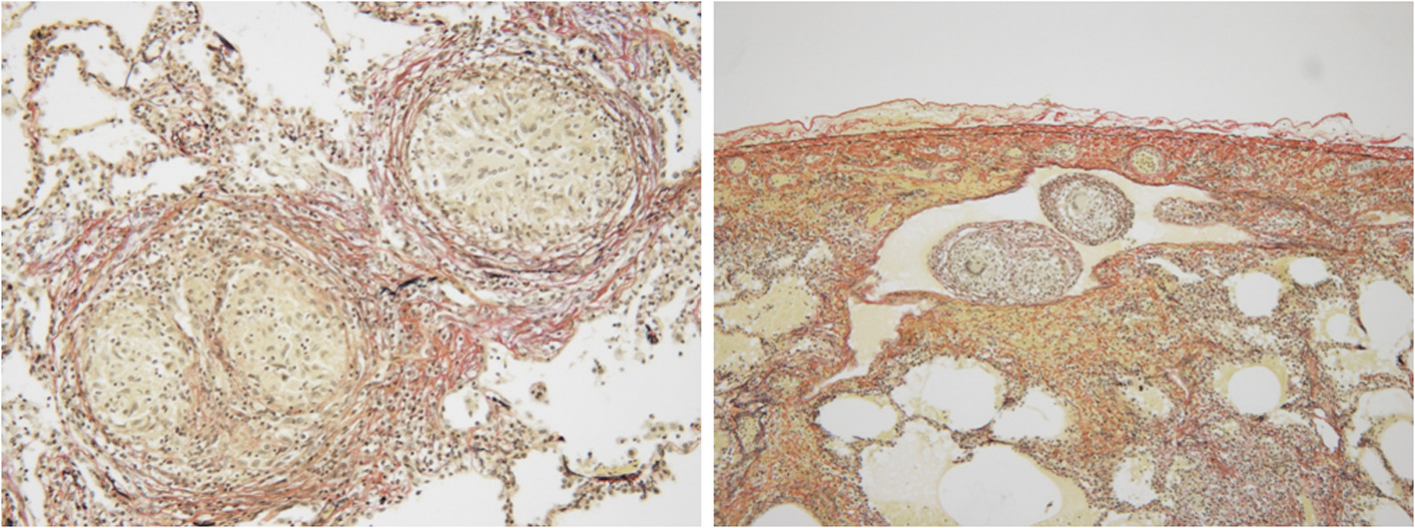
**The surgical specimen of a mass-like opacity in the left lower lobe.** Small, loose-formed, non-necrotizing granulomas are seen in the bronchiole and alveolar duct (left) and in the lymph tract just below the pleura (right); no granulomatous vasculitis is seen. The findings are consistent with hypersensitivity pneumonitis with poor drainage of a large amount of granulomas (Elastica van Gieson stain, left ×200, right ×100).

On admission, his oxygen saturation at rest was 92%, and there were end-inspiratory crackles on auscultation of both lung bases. Results of laboratory tests showed a white blood cell count of 11,380/mm^3^, C-reactive protein of 4.67mg/dL, erythrocyte sedimentation rate of 37mm after 1 hour, and a serum KL-6 of 3824U/mL. Lactase dehydrogenase and angiotensin-conversion enzyme levels were within the normal range. Screening for cytomegalovirus antigen and QuantiFERON®-TB Gold (Cellestis Limited, Carnegie, Victoria, Australia) were negative, as were rheumatoid factor, antineutrophilic cytoplasmic antibody, antinuclear antibody and anti-Jo-1 antibody. Pulmonary function studies showed that first-second vital capacity was 2.68L (86.2% predicted), a vital capacity of 3.25L (72.5% predicted), a ratio of residual volume to total lung capacity of 25.8%, and a single-breath carbon monoxide diffusing capacity of 64.5% predicted. The serum precipitins reaction test was 2+ for *Aspergillus* species except *Aspergillus niger* and negative for *Trichosporon asahii*. A chest radiograph showed bilateral fine reticulonodular shadow in his lower lung areas. Computed tomography of his chest revealed bilateral diffuse fine centrilobular nodules and some mass lesions in lower lobes that do not fit a common image of hypersensitivity pneumonitis, but no lesions in interstitial areas (Figure 1). Bronchoalveolar lavage fluid from his right middle lobe consisted of 37% macrophages and 62% T cells, with a CD4 to CD8 ratio of 0.84. Fungal and mycobacterial cultures of bronchoalveolar lavage fluid were negative, as were polymerase chain reactions for tuberculosis, nontuberculous *Mycobacterium*, and *Pneumocystis jirovecii*. A transbronchial lung biopsy from his right middle lobe demonstrated small, loosely formed, non-necrotizing granulomas surrounded by slight collagenous fibers with or without giant cells in the bronchioles and alveolar duct, as well as lymphocytic alveolitis. These features were consistent with hypersensitivity pneumonitis.The patient stopped working at the warehouse, and his oxygen saturation returned to normal in 3 weeks; however, centrilobular nodules persisted on computed tomography, the mass lesions enlarged, and his serum KL-6 was still high at 4000U/mL. As a diagnostic procedure, a surgical biopsy of a mass lesion was performed. The specimen revealed massive granulomas spreading not only in alveolar ducts, as in the transbronchial lung biopsy findings, but also in the lymph tract just below the pleura. Granulomatous vasculitis was not evident, and these findings were consistent with poor drainage of a large amount of granulomas (Figure 2).

Considering the negative findings for infectious disease, high-dose steroid therapy was started. The centrilobular nodules then disappeared, the mass lesions shrank, and his serum KL-6 decreased.

## Discussion

According to the analysis of 25 CGD surgical specimens, granulomas in patients with CGD were typically 100μm or less in diameter and contained central neutrophil microabcesses surrounded by a layer of palisading epithelioid histiocytes and giant cells. They spread not only in airways, but also in lung parenchyma. In the same specimens, *Aspergillus* species were isolated in 44% and bacterial organisms were cultured in 28% [3,4].

In this case, the following three differential diagnoses of mass-like opacities were considered: CGD, fungal or bacterial infection, and hypersensitivity pneumonitis. First, the granulomas were loosely formed and relatively larger than those of CGD, they did not contain central microabcesses, and they spread through the airway to the lymph tract, consistent with hypersensitivity pneumonitis. Second, bacterial or fungal infection was proven neither histologically nor bacteriologically. Although no inhaled antigen was proven, his past history of working at a warehouse indicated that fungal exposure might have caused hypersensitivity pneumonitis.

Recent reports show an excessive release of inflammatory cytokines such as from neutrophils in patients with CGD [5]. Animal models of CGD also show the hyperinflamed immune dysregulation seen in CGD, which explain excessive granuloma formation in organs such as the gastrointestinal system and the genitourinary system [6]. Experiments performed in this case showed that the patient’s T cells in the bronchoalveolar lavage fluid produced excessive cytokines after incubation with or without antigen stimulation *in vitro* (data not shown). These findings lead to the possibility that excessive inflammation in T cells of patients with CGD explains atypical granuloma formation, such as the homogeneous and excessive amount of loose-formed granulomas through the airway spreading into the lymph tracts, despite the subacute clinical course.

## Conclusions

When we encounter a case of hypersensitive pneumonitis showing atypical pulmonary mass-like opacities in a patient with CGD, we should consider hyperinflammatory status and excessive granuloma formation of CGD and start with high-dose steroid therapy as treatment.

## Consent

Written informed consent was obtained from the patient for publication of this case report and accompanying images. A copy of the written consent is available for review by the Editor-in-Chief of this journal.

## Competing interests

The authors declare that they have no competing interests.

## Authors’ contributions

SK performed the histological examination of the lung. TK and MO carried out the laboratory experiments with the patient’s T cells. MH and HS provided diagnostic and therapeutic suggestions. YK was a major contributor in writing the manuscript. All authors read and approved the final manuscript.
